# Serum neuronal exosomes predict and differentiate Parkinson’s disease from atypical parkinsonism

**DOI:** 10.1136/jnnp-2019-322588

**Published:** 2020-04-09

**Authors:** Cheng Jiang, Franziska Hopfner, Antigoni Katsikoudi, Robert Hein, Candan Catli, Samuel Evetts, Yongzhi Huang, Hong Wang, John W Ryder, Gregor Kuhlenbaeumer, Guenther Deuschl, Alessandro Padovani, Daniela Berg, Barbara Borroni, Michele T Hu, Jason J Davis, George K Tofaris

**Affiliations:** 1 Nuffield Department of Clinical Neurosciences, University of Oxford, John Radcliffe Hospital, Oxford, UK; 2 Department of Neurology, Christian-Albrechts-University, Kiel, Germany; 3 Department of Chemistry, Physical and Theoretical Chemistry Laboratory, University of Oxford, Oxford, United Kingdom; 4 Oxford Parkinson’s Disease Centre, Oxford, United Kingdom; 5 Nuffield Department of Surgical Sciences, University of Oxford, John Radcliffe Hospital, Oxford, United Kingdom; 6 Lilly Research Laboratories, Eli Lilly and Company, Indianapolis, Indiana, USA; 7 Department of Clinical and Experimental Sciences, Neurology Unit, University of Brescia, Brescia, Italy

## Abstract

**Objective:**

Parkinson’s disease is characterised neuropathologically by α-synuclein aggregation. Currently, there is no blood test to predict the underlying pathology or distinguish Parkinson’s from atypical parkinsonian syndromes. We assessed the clinical utility of serum neuronal exosomes as biomarkers across the spectrum of Parkinson’s disease, multiple system atrophy and other proteinopathies.

**Methods:**

We performed a cross-sectional study of 664 serum samples from the Oxford, Kiel and Brescia cohorts consisting of individuals with rapid eye movement sleep behavioural disorder, Parkinson’s disease, dementia with Lewy bodies, multiple system atrophy, frontotemporal dementia, progressive supranuclear palsy, corticobasal syndrome and controls. Longitudinal samples were analysed from Parkinson’s and control individuals. We developed poly(carboxybetaine-methacrylate) coated beads to isolate L1 cell adhesion molecule (L1CAM)-positive extracellular vesicles with characteristics of exosomes and used mass spectrometry or multiplexed electrochemiluminescence to measure exosomal proteins.

**Results:**

Mean neuron-derived exosomal α-synuclein was increased by twofold in prodromal and clinical Parkinson’s disease when compared with multiple system atrophy, controls or other neurodegenerative diseases. With 314 subjects in the training group and 105 in the validation group, exosomal α-synuclein exhibited a consistent performance (AUC=0.86) in separating clinical Parkinson’s disease from controls across populations. Exosomal clusterin was elevated in subjects with non-α-synuclein proteinopathies. Combined neuron-derived exosomal α-synuclein and clusterin measurement predicted Parkinson’s disease from other proteinopathies with AUC=0.98 and from multiple system atrophy with AUC=0.94. Longitudinal sample analysis showed that exosomal α-synuclein remains stably elevated with Parkinson’s disease progression.

**Conclusions:**

Increased α-synuclein egress in serum neuronal exosomes precedes the diagnosis of Parkinson’s disease, persists with disease progression and in combination with clusterin predicts and differentiates Parkinson’s disease from atypical parkinsonism.

## Introduction

Parkinson’s disease (PD) is the most common movement disorder with a long prodromal phase and risk of progression to dementia. Disease progression broadly correlates with the evolution of Lewy body and neuritic pathology,^1^ which involves the intraneuronal accumulation and aggregation of α-synuclein.^2^ In contrast, predominantly oligodendroglial α-synuclein accumulation is the primary neuropathological feature of multiple system atrophy (MSA), a life-limiting condition characterised by extrapyramidal, cerebellar and autonomic symptoms.^2^ Accurate measurements of neuron-associated α-synuclein could be a useful biomarker in the prediction or differential diagnosis of PD and especially attractive for the objective assessment of novel drugs currently in clinical trials, that lower α-synuclein for disease modification. Although cerebrospinal fluid (CSF) total α-synuclein is reduced in patients with PD compared with controls,^3^ meta-analyses showed an unsatisfactory diagnostic accuracy with a pooled sensitivity between 78% and 88% and a specificity between 40% and 57%.^4^

Currently, there is no blood test in clinical practice that can either predict risk or reliably distinguish PD from atypical parkinsonian syndromes. α-Synuclein is present in peripheral fluids^5^ but its concentration in blood is strongly influenced by red blood cells, which are the source of >99% of the protein.^6^ For this reason, blood content of free total α-synuclein in PD patients is of limited utility,^7^ partly due to contamination with red cell haemolysis. An alternative approach is the quantification in blood of the fraction of α-synuclein that is released from neuronal tissues such as α-synuclein in neuron-derived extracellular vesicles. Exosomes are endosome-derived small extracellular vesicles (40–120 nm) released by most cell types including neurons.^7^ Circulating exosome composition and function is altered in PD^8^ and neuron-derived exosomes isolated from plasma contain disease-associated proteins, including α-synuclein.^9^

A major challenge in translating neuron-specific exosomal α-synuclein measurements in blood into a clinically useful test is the separation of exosome subpopulations in complex biological fluids from contaminants, including α-synuclein derived from peripheral sources. Previous studies in a small number of patients or single cohorts have yielded variable results and overall low performance.^9 10^ In this study, we generated an improved assay to immunocapture serum neuronal exosomes and assess their clinical utility across the spectrum of Parkinson’s pathology and atypical parkinsonian syndromes.

## Materials and methods

### Patient populations

A total of 664 subjects were included in this study (table 1). Serum samples and clinical data were collected from patients with polysomnographically confirmed rapid eye movement sleep behaviour disorder (RBD) (n=65), PD (n=275), dementia with Lewy bodies (DLB) (n=21), MSA (n=14), frontotemporal dementia including the behaviour variant or primary progressive aphasia (FTD, n=65), progressive supranuclear gaze palsy (PSP) (n=35) and corticobasal syndrome (CBS) (n=45) and healthy controls (HC, n=144,). Patients and controls were recruited from three different centres: The Oxford Parkinson’s Disease Centre Discovery cohort is a prospective cohort of recently diagnosed patients with PD who were recruited from 11 hospitals in the Thames Valley region between September 2010 and January 2016. Full details of the discovery cohort and full inclusion/exclusion criteria have been published elsewhere.^11^ MSA patients were recruited from a specialist MSA clinic in Oxford as part of the Discovery cohort. The Kiel-PD cohort is a cross-sectional cohort of patients with PD and HC recruited at the Department of Neurology, Christian-Albrechts-University Kiel, Germany between 2013 and 2019. Full details of the Kiel-PD cohort were published previously.^12^ Longitudinal samples were collected from the Oxford Cohort in three follow-up visits for PD patients over a period of 40.3±8.5 months and two visits for controls 18 months apart. Patients with PD, DLB, FTD, PSP and CBS were also recruited at the Centre for Neurodegenerative Disorders and the Centre for Movement Disorders, Neurology Unit, University of Brescia, Italy. Full details of the Brescia cohort and inclusion/exclusion criteria have been published elsewhere.^13^ Diagnostic criteria were uniformly applied across cohorts for PD,^14^ probable PSP^15^ CBS,^16^ MSA,^17^ DLB^18^ and FTD.^19 20^ Demographic comparisons are shown in online supplementary table 1. We also used sera from neuropathologically confirmed cases of DLB with relatively pure α-synuclein pathology (n=10) and HC (n=10) that were recruited as part of the OPTIMA study. These patient studies were approved by the corresponding local Hospital Ethics Committees. Written informed consent was obtained from all participants or their caregivers.

10.1136/jnnp-2019-322588.supp1 Supplementary data

**Table 1:** Summary of individual cohort characteristics and concentrations of exosome markers

	RBD	PD	PDD	DLB	MSA	HC	FTD	PSP	CBS
Oxford	No of individuals	65	48	26	10*	14	31	–	–	–
	Male (female)	62(3)	36(12)	21(5)	7 (3)	10(4)	22(9)	–	–	–
	Age	64.2±8.3	62.8±9.3	70.2±6.6	82.8±7.7	68.1±10.8	66.3±8.8	–	–	–
	Duration of disease (years)	na	1.8±2.1	3.5±4	–	4.9±2.6	na	–	–	–
	UPDRS	5.1	32.9	39.6	–	27.7	na	–	–	–
	MoCA	25.5	27.1	18.2	–	27.3	na	–	–	–
	exo α-synuclein (pg/mL)	26.69±12.8	22.36±9.5	25.34±10.6	17.23±4.6	10.72±4.5	12.48±5.1	–	–	–
	exo Clusterin (ng/mL)	10.01±5.2	7.85±3.6	9.56±4	6.99±3	6.84±3.2	11.25±2.6	–	–	–
	exo Syntenin-1 (ng/mL)	32.85±27.1	43.31±21.6	38.29±22	28.10±10.5	14.77±5.8	33.40±15.9	–	–	–
Kiel	No of individuals	–	155	15	–	–	113	–	–	–
	Male (female)	–	96(59)	11(4)	–	–	72(41)	–	–	–
	Age	–	67.5±9.3	73.9±8.2	–	–	59.0±4.8	–	–	–
	Duration of disease (years)	–	9.30±6.1	14.4±6.6	–	–	na	–	–	–
	UPDRS	–	23.44	39.2	–	–	na	–	–	–
	MoCA	–	27.54	18.47	–	–	na	–	–	–
	exo α-synuclein (pg/mL)	–	29.32±20.5	36.57±24.4	–	–	12.72±6.1	–	–	–
	exo Clusterin (ng/mL)	–	10.60±6.4	12.77±5.9	–	–	8.08±5.2	–	–	–
	exo Syntenin-1 (ng/mL)	–	25.65±20.3	38.82±22.5	–	–	18.81±12	–	–	–
Brescia	No of individuals	–	27	4	11	–	–	65	35	45
	Male (female)	–	17(10)	2 (2)	7 (4)	–	–	38(27)	18(17)	27(18)
	Age	–	65.0±9.4	71.0±15.8	68.6±4.9	–	–	62.5±7	68.0±7.5	61.1±7.2
	Duration of disease (years)	–	na	18.5±5.8	3.4±3.0	–	–	2.9±2.5	2.8±1.8	1.9±1.3
	UPDRS	–	20.11	39.75	na	–	–	na	24.48	22.30
	MoCA	–	26.78	16.50	na	–	–	na	21.40	22.49
	exo α-synuclein (pg/mL)	–	25.61±19	20.96±5.4	16.87±3.1	–	–	12.60±4	9.20±4.9	9.93±3.7
	exo Clusterin (ng/mL)	–	7.56±5.8	6.39±4.8	6.15±5.2	–	–	22.22±10.5	18.42±8.8	16.16±6.1
	exo syntenin-1 (ng/mL)	–	22.95±10	15.92±3.9	20.05±5.3	–	–	20.81±20.9	44.31±23	54.73±25

Data represent the mean at the time of sample collection. UPDRS and MoCA were available in 48% of HC.

*Post-mortem cases

CBS, corticobasal syndrome; DLB, dementia with Lewy bodies; FTD, frontotemporal dementia; HC, healthy controls; MoCA, Montreal cognitive assessment; MSA, multiple system atrophy; na, not applicable; PD, Parkinson’s disease; PDD, Parkinson’s disease with dementia; PSP, progressive supranuclear gaze palsy; RBD, rapid eye movement sleep behaviour disorder; UPDRS, Unified Parkinson's Disease Rating Scale.

### Exosome immunocapture

Blood samples from the three centres were collected during the patient assessment, the serum was isolated, aliquoted and frozen at −80°C until further use. All samples were sent on dry ice and processed in a blinded fashion at Oxford. All samples went only one freeze-thaw cycle. For exosome isolation a three-step sequential spin (300 g for 10 min, 2000 g for 20 min and 10 000 g for 30 min) was used to remove cellular debris, proteins aggregates and fatty material in the serum. 0.5 mL of supernatant, that is, precleared serum, was transferred to protein low-binding tubes (Eppendorf) for immunocapture using anti-L1CAM antibodies (ab80832, Abcam) preconjugated to poly(carboxybetaine methacrylate) (pCBMA) coated beads that were generated to reduce non-specific adsorption (see online supplementary figures 1–4) for assay development). The immunobeads were incubated at 4°C overnight on a rotating mixer and bead-exosomes complexes were collected by magnetic separation and washed successively with 0.05% Tween-20 in PBS and PBS. Isolated exosomes were lysed in 1% triton X-100 in PBS with 4% protease inhibitors for 15 min at room temperature for exosomal protein quantification. Exosomes were extracted in batches with disease and control samples distributed into each batch, followed by sample blinding. Although we did not quantify enrichment for neuronal proteins in our preparations, our mass spectrometry analysis and immunoblotting confirmed the presence of neural cell adhesion molecule and L1CAM in immunocaptured exosomes as shown in online supplementary figure 3. Our isolation protocol and its validation followed MISEV2018 recommendations.^21^ Based on the detection of syntenin-1 and tsg101 in vesicle lysates (online supplementary figure 3C) and estimated average size around 100 nm by nanoparticle tracking analysis (online supplementary figure 4), our preparations consist of extracellular vesicles with characteristics of exosomes, herein referred to as exosomes

### Detection of exosomal proteins

Electrochemiluminescence (ECL) was performed in 96-well Meso Scale Discovery (MSD) U-Plex plates that enable multiplexing of markers in the same exosome preparation. All steps were performed at room temperature. Three unique linkers for the selected markers (syntenin-1, clusterin and α-synuclein) were used according to the manufacturer’s protocol. After three washes, detection antibodies with Sulfo-TAG-labelling were incubated for 1 hour. Following washes and addition of MSD Read buffer the plates were read using the MSD-ECL platform (QuickPlex SQ 120) and data were analysed with the MSD Discovery Workbench 3.0 Data Analysis Toolbox. Antibody pairs for clusterin and α-synuclein were provided by MSD and preconjugated with biotin and ruthenium tag. Additive-free anti-syntenin-1 goat polyclonal antibody (PAB7132, Abnova) and anti-syntenin-1 rabbit monoclonal antibody (ab236071, Abcam) were conjugated with biotin and ruthenium and used as capture and detection antibodies, respectively. For phosphorylated α-synuclein at serine129 (pSer129) detection, the antibody pair used consists of a biotinylated antibody against pSer129 α-synuclein (11A5, purified from PTA-8222 hybridoma cell line, American type culture collection) acting as capture antibody and a ruthenium labelled antibody against total α-synuclein (4B12, Biolegend) acting as the detection antibody. For combined exosomal α-synuclein, clusterin and syntenin-1 we developed triplex MSD and demonstrated specific detection of these markers in immunocaptured exosomes (online supplementary figure 5) and consistency of the test when the same sample was tested on two separate occasions 6 months apart (online supplementary figure 6). For all assays, we assessed dynamic range and lower limit of detection (online supplementary figures 7,8).

### Statistical analysis

For multiple comparisons, we performed non-parametric statistical testing as the data were not normally distributed (Kruskal-Wallis one-way analysis of variance with the Dunn test for post hoc comparison between individual pairings) using Prism 8 Graphpad (San Diego, USA). Relationships between exosome markers and disease duration, gender, Montreal cognitive assessment (MoCA) scores and UPDRS motor scores were analysed with bivariate correlation using Pearson’s correlation coefficients. To assess the performance of the proposed biomarker in separating α-synucleinopathies from controls and define cut-off values, we used the Kiel and Brescia cohorts as a training group (n=314) and the Oxford cohort as a validation group (n=105). Data from these groups were analysed using receiver operating characteristic. The ‘optimum’ cut-off point was determined by Youden’s index, that is, the value associated with the maximal value of sensitivity +specificity−1. Values with p<0.05 were regarded as significant. Logistic regression analysis was used to determine the best combination of different protein markers (clusterin and α-synuclein) for discriminating between diagnostic groups or sets of subgroups. Longitudinal samples were analysed using linear mixed model to investigate the correlation between biomarker concentration and duration, with sample at first visit treated as baseline. The robust regression and outlier removal method was applied to test for outliers. Logistic regression and linear mixed model were performed using MATLAB (MATLAB and Statistics Toolbox Release 2014a The MathWorks, Natick, Massachusetts, USA).

### Data availability

Anonymised individual participant data and the study protocol will be shared with qualified parties on request to the corresponding author.

## Results

### Neuron-derived exosomal α-synuclein is increased across the spectrum of Lewy body diseases

**Figure 1:**
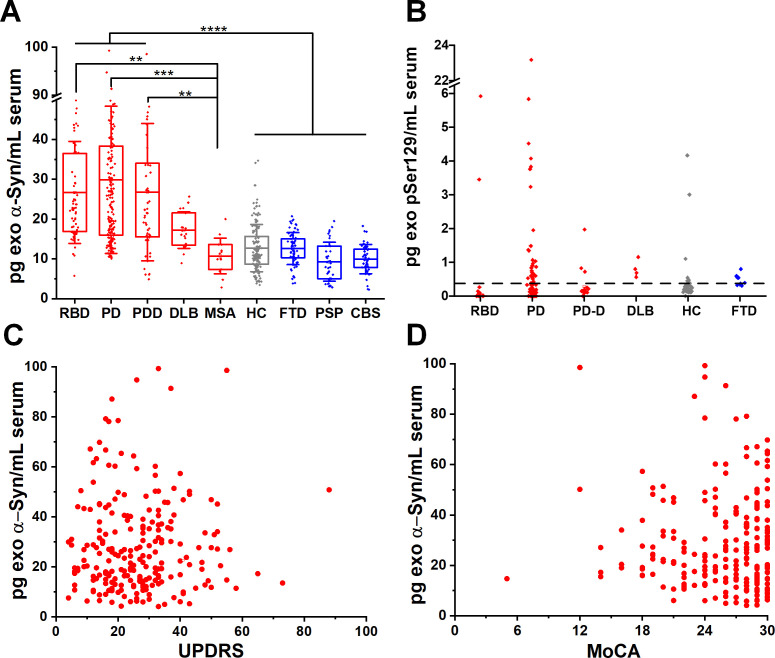
Neuron-derived exosomal α-synuclein is increased across the spectrum of Lewy body pathology. (A) Boxplots of mean total α-synuclein across the spectrum of conditions with Lewy body pathology (RBD, motor PD, PDD, DLB), MSA and unrelated neurodegenerative diseases (FTD, PSP, CBS) as well as age-matched and sex-matched controls. Twofold increase in the content of α-synuclein was detected in L1CAM-positive exosomes isolated from conditions characterised by Lewy body pathology. (B) At the lowest detectable concentration (0.32 pg/mL), pSer129 α-synuclein was detected in a subgroup of PD patients that were tested (55.8%). No significant correlation was seen between total exosomal α-synuclein and either UPDRS (C), r=0.0267) or MoCA (D), r=0.0621) in PD patient samples. **P<0.01, ***P<0.001, ****P<0.0001. Mean values with IQR of exosomal markers and whisker range using SD with coefficient of 1 were used in the boxplots. CBS, corticobasal syndrome; DLB, dementia with Lewy bodies; FTD, frontotemporal dementia; PD, Parkinson’s disease; PDD, Parkinson’s disease with dementia; RBD, rapid eye movement sleep behaviour disorder.

We blindly analysed serum samples from 664 subjects across the spectrum of Lewy body pathology by assaying patients in the prodromal, motor and dementing stage. To this end, we separated the PD participants according to MoCA scores, corrected for education into those with pure motor PD or PD with cognitive impairment. Cognitive impairment within the PD cohorts was defined as MoCA screening score of less than 21/30^22^ at the time of sample collection. Thus, we subsequently analysed blindly subgroups of motor PD (n=230) or PD with cognitive impairment referred to herein as PD dementia, PDD (n=45). We also included a group of 21 cases with the clinical diagnosis of DLB, 10 of which were confirmed at autopsy. A group of idiopathic RBD without motor signs (n=65) was also used as a surrogate of prodromal PD as prospective cohort studies have observed a very strong association between RBD and subsequent clinically defined α-synucleinopathy, with up to 80% of cases converting primarily to PD or DLB.^23 24^ We found that α-synuclein was elevated in RBD, PD and DLB exosomes by ~twofold compared with controls MSA or other proteinopathies (figure 1A and table 1). Specifically, α-synuclein content in L1CAM-positive exosomes is similarly elevated (data shown as mean±SD) in RBD (26.69±12.82 pg/mL), motor PD (27.44±18.82 pg/mL) and PD with dementia (PDD 26.76±17.25 pg/mL) when compared with healthy subjects (HC, 12.71±5.93 pg/mL). α-Synuclein was also elevated in DLB (17.23±4.58 pg/mL). We demonstrated the association between increased release of α-synuclein in neuronal exosomes and Lewy body pathology by testing sera taken pre-mortem in autopsy confirmed control and DLB cases (n=10 per group). In these two subgroups, mean neuronal exosome-associated α-synuclein was 17.60±5.86 pg/mL in DLB and 10.50±4.60 pg/mL in controls (1.7-fold increase, p=0.0097). As expected, exosomal α-synuclein concentration was much lower compared with reported levels of free total α-synuclein in blood (10–17 ng/mL).^3 9^ Interestingly, neuron-derived exosomal α-synuclein was not elevated in any of the cases with MSA (10.72±4.49 pg/mL), a disease characterised primarily by oligodendroglial pathology, despite the fact that MSA samples were collected and processed using an identical procedure to PD samples.

To assess the abundance of neuron-derived exosomal α-synuclein in unrelated neurodegenerative diseases, we included patients with FTD (n=65) which is pathologically characterised primarily by tau or TAR DNA binding protein 43 (TDP-43) aggregation, and patients with PSP (n=35) and CBS (n=45) who present with atypical parkinsonism and pathologically are characterised by fibrillar aggregates of four repeat tau. We found that α-synuclein content in L1CAM-positive exosomes from these diseases is similar to HC as shown in figure 1A (FTD, 12.60±4.03 pg/mL; PSP, 9.20±4.90 pg/mL; CBS, 9.93±3.68 pg/mL).

In 226 subjects (18 RBD, 77 PD, 36 PDD, 11 DLB, 69 HC, 15 FTD), we also asked whether elevated α-synuclein in Lewy body disease is phosphorylated at serine 129 (pSer129) in L1CAM-positive exosomes and has a value as a blood-based biomarker. pSer129 α-synuclein is the main disease-associated modification that accounts for more than 90% of α-synuclein found in Lewy bodies.^25^ With an estimated cut-off value of 0.32 pg/mL for the lowest detectable concentration for this assay (online supplementary figure 8), pSer129 α-synuclein was elevated in a subgroup of 43 patients with PD (55.8% of total PD tested, figure 1B). In this PD subpopulation, pSer129 α-synuclein did not correlate with UPDRS (r=0.2149, p=0.1717) or MoCA (r=0.0081, p=0.9591). Unlike previous studies,^9 10^ we did not detect any significant correlation between total exosomal α-synuclein and either UPDRS (r=0.0267) or MoCA (r=0.0621) as shown in figure 1C, D.

### α-Synuclein in serum neuronal exosomes from Parkinson’s patients is consistently elevated across cohorts

**Figure 2:**
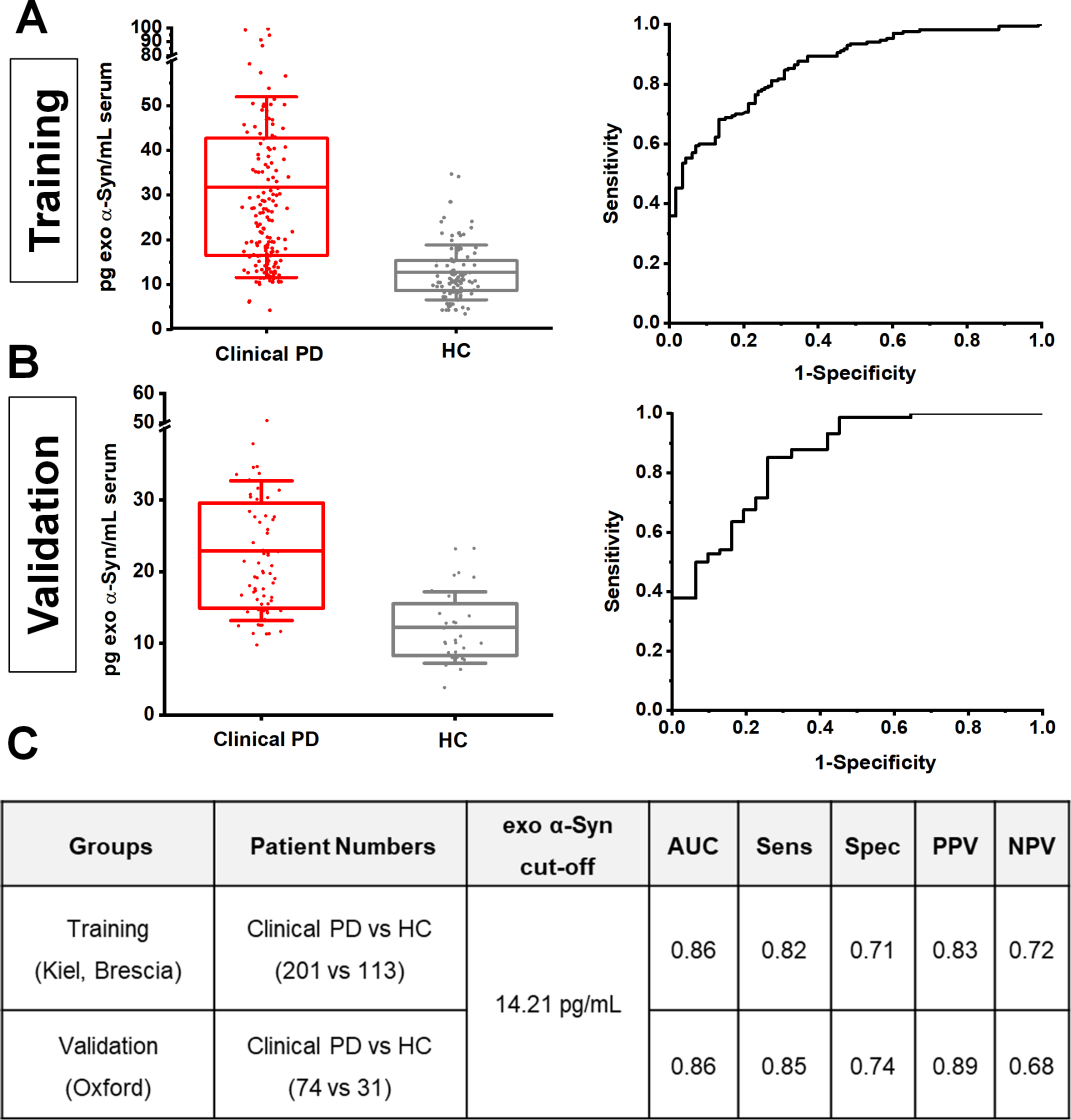
Estimation of cut-off values of neuronal exosome-associated α-synuclein between cohorts. Boxplots of mean exosomal α-synuclein levels and corresponding ROC curves in training (A) and validation groups (B). When an exosomal α-synuclein cut-off value ≥14.21 pg/mL estimated from the training group (Keil and Brescia) was applied to the validation group (Oxford), assay performance analysis revealed a consistent result across populations with similar area under a curve (AUC), sensitivity (senS), specificity (spec), positive (PPV) and negative (NPV) predictive values in distinguishing clinical PD from controls as shown in panel C). HC, healthy controls; PD, Parkinson’s disease; ROC, receiver operating characteristic.

To further assess the consistency of neuronal exosome-associated α-synuclein in differentiating clinical PD from healthy subjects across populations, we applied a two-stage design model: a training group of 314 subjects from the Kiel and Brescia cohorts was used to identify an optimal cut-off value, which was then applied to an independent validation group of 105 subjects from the Oxford cohort. This revealed that at 14.21 pg/mL, the assay exhibits a consistent performance (training vs validation) with an area under the curve of 0.86, sensitivity of 0.82 vs 0.85, specificity of 0.71 vs 0.74, and positive predictive value of 0.83 vs 0.89 and negative predictive value of 0.72 vs 0.68 as shown in figure 2.

### α-Synuclein and clusterin measurement improved the predictive value of the exosome test

**Figure 3:**
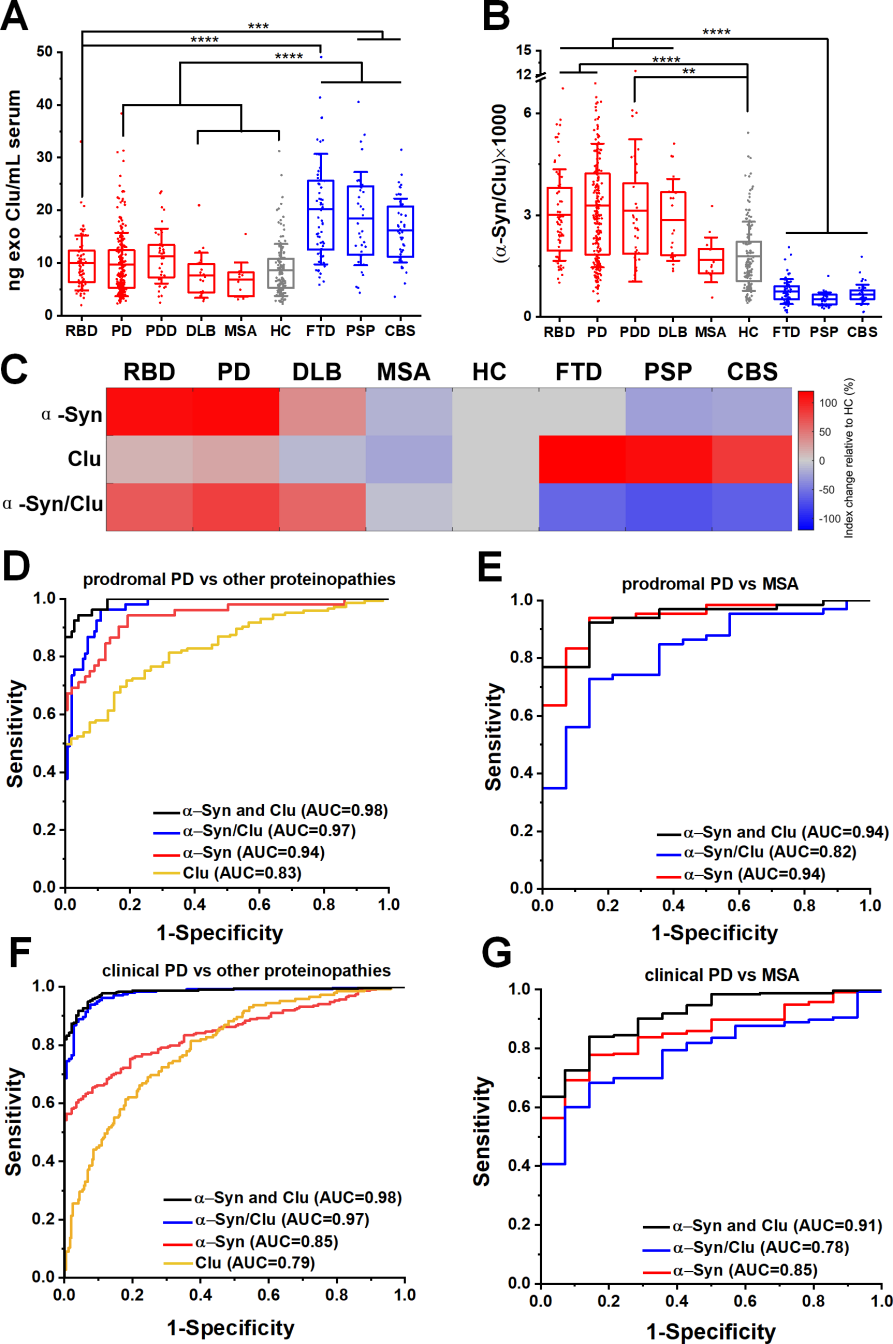
Neuron-derived exosomal clusterin is increased in tauopathies and when combined with α-synuclein improved the differential diagnosis. (A) Clusterin (CLU) release in serum neuronal exosomes is increased in FTD, PSP and CBS but not RBD, PD, PDD, DLB, MSA or age-matched and sex-matched controls. (B) Ratio of α-synuclein to clusterin improved the separation between Lewy body pathology and alternative proteinopathies. (C) Heatmap illustration of exosome profiles using α-Syn, Clu or α-Syn/Clu differentiating between diseases. The change in the concentration of each exosome marker was normalised to the value of HC. ROC analysis of individual markers and their ratio or linear regression analysis of composite measurements revealed an additive effect of the two biomarkers in differentiating prodromal or clinical PD from alternative proteinopathies as shown in (D, F) or MSA as shown in (E, G) Clinical PD refers to the combined group of PD and PDD. **P<0.01, ***P<0.001, ****P<0.0001. AUC, area under a curve; CBS, corticobasal syndrome; DLB, dementia with Lewy bodies; FTD, frontotemporal dementia; HC, healthy controls; MSA, multiple system atrophy; PD, Parkinson’s disease; PDD, Parkinson’s disease with dementia; PSP, progressive supranuclear gaze palsy; RBD, rapid eye movement sleep behaviour disorder; ROC, receiver operating characteristic.

Our mass spectrometry analysis identified clusterin as an abundant exosome-associated protein (online supplementary figure 3E), which is a known risk gene^26 27^ for Alzheimer’s dementia and was shown to interact with TDP-43 and tau in functional studies.^28^ We, therefore, hypothesised that the quantification of clusterin in neuronal exosomes may aid the stratification of patients. We found that clusterin was elevated in FTD (20.22±10.47 ng/mL), PSP (18.42±8.84 ng/mL) and CBS (16.16±6.07 ng/mL) (figure 3A) but not elevated in RBD (10.01±5.22 ng/mL), clinical PD (9.72±6.02 ng/mL), MSA (6.84±3.24 ng/mL) or HC (8.67±4.92 ng/mL). The differential abundance of clusterin in unrelated proteinopathies suggests that integration of clusterin in a blood-based exosome test could be of value in distinguishing PD patients from tau-related atypical parkinsonian syndromes (figure 3B). This is demonstrated in the heatmap (figure 3C) that summarises the overall trend of the biomarkers (mean concentrations were used) across different patient groups when normalised to HC. In contrast, the generic exosomal protein syntenin-1 did not exhibit a disease-specific distribution with sufficient separation to contribute as a biomarker (online supplementary figure 9).

To further evaluate the clinical potential of combined α-synuclein and clusterin measurements in L1CAM-positive exosomes as biomarkers, we assessed α-synuclein to clusterin ratio and applied a logistic regression model for the combination of these markers across all cohorts. The composite α-synuclein and clusterin measurement exhibited an improved AUC, sensitivity and specificity estimates for differential diagnosis in predicting clinical PD versus other proteinopathies, with an AUC=0.98 (sensitivity 0.94; specificity 0.96), even in the prodromal phase of PD (RBD vs other proteinopathies, AUC=0.98, sensitivity 0.95, specificity 0.93) as shown in figure 3D, F and online supplementary table 2. This measurement also exhibited a high performance in distinguishing prodromal or clinical PD from MSA (AUC=0.94 and 0.91 respectively) as summarised in figure 3E, G.

### Longitudinal trajectories of exosomal α-synuclein and clusterin with disease progression

**Figure 4:**
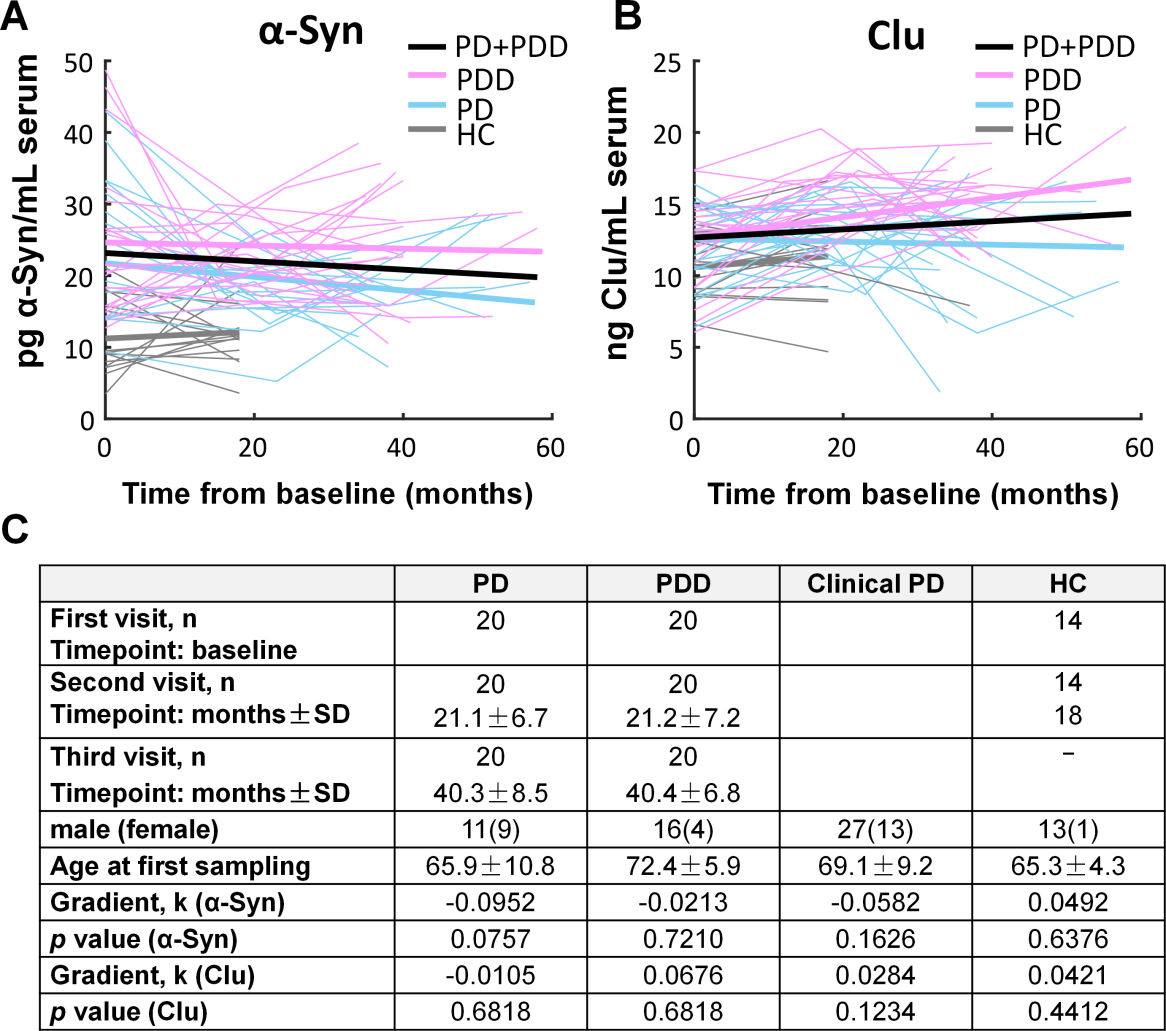
Longitudinal analysis of exosome-associated α-synuclein and clusterin. Linear mixed model of exosomal α-synuclein (A) and clusterin (B) was fitted to the longitudinal values with time from first sampling as a covariant, and patients stratified by level at initial visit in relation to median value. Persistent separation between disease subgroups and controls but no overall significant difference in the gradient from zero was identified when comparing α-synuclein in clinical PD to control samples. Clinical PD refers to the combined group of PD and PDD. Patient characteristics and p values are summarised in C. HC, healthy controls; PD, Parkinson’s disease; PDD, Parkinson’s disease with dementia.

To investigate the variability of neuron-associated exosomal markers within an individual over the course of the disease, we blindly analysed prospective longitudinal samples from the Oxford cohort. A linear mixed model was applied to fit the longitudinal values of neuron-derived exosomal α-synuclein and clusterin with time from first sampling as a covariant, and patients stratified by level at initial visit in relation to median value. Longitudinal sample numbers for PD, PDD and controls are summarised in figure 4. Overall, the gradient did not differ significantly from zero for either stratum of α-synuclein or clusterin when comparing clinical PD (PD or PDD or combination) or controls. This analysis indicates that neuron-derived exosomal α-synuclein levels remain elevated within individuals with PD in three subsequent visits spanning over a period of 40.3±8.5 months from initial sampling with persistent separation from controls as shown in figure 4.

## Discussion

Our results show for the first time that as a single cross-sectional measurement, serum neuronal exosome-associated α-synuclein and clusterin performs best as a predictive marker of PD versus atypical parkinsonism including MSA in clinical and prodromal PD, outperforming any previously reported blood-based assay or CSF total or pathogenic α-synuclein.^3 29^ This enhanced performance of our serum neuronal exosome test across samples collected at multiple sites is at least in part due to our improved immunocapture assay using zwitterionic coating, a surface that resists non-specific binding.^30^ Although exosomal α-synuclein per se was not sufficiently sensitive and specific to be used as a diagnostic marker, we show that this readout is consistent when tested across PD populations from three countries and stable over disease progression when assessed within individuals suggesting that it could be considered as a pharmacodynamic biomarker for α-synuclein targeting therapies in PD. Together with previous work in Alzheimer’s disease (AD),^31 32^ our findings demonstrate that protein cargoes in L1CAM-positive extracellular vesicles exhibit distinct composition in neurodegenerative diseases that predate the clinical phase and offer a promising means to develop blood-based predictive markers of the relevant brain pathology.

Our finding of increased neuronal exosome α-synuclein levels in PD and PDD compared with controls by ~2fold is broadly in line with a previously reported twofold increase in motor PD compared with controls when tested in smaller patient groups or single cohorts.^9 10^ This consistent change across three studies firmly establishes that increased exosomal α-synuclein is a validated disease-relevant observation in PD. Unlike previous studies, our data indicate that neuron-derived exosomal α-synuclein content does not correlate with clinical parameters when assessed in larger number of patients across cohorts. Instead, we found that neuronal exosome α-synuclein levels are elevated early in the disease process as exemplified in patients with RBD, a group at high risk of developing PD, and remain elevated with disease progression. These findings regarding α-synuclein in neuronal exosomes would be consistent with neuropathological studies, showing that the average number of dopaminergic neurons with Lewy bodies is stable with disease progression^33^ and diffuse α-synuclein pathology does not always correlate with clinical symptoms.^34 35^ We confirmed the association between serum neuronal exosome content and brain Lewy pathology in cases with postmortem diagnosis of diffuse Lewy body disease. Interestingly, neuron-derived exosomes from MSA serum did not exhibit a similarly increased α-synuclein despite the fact that the samples were collected and processed using identical procedures. One explanation is that α-synuclein in MSA accumulates primarily in oligodendrocytes and therefore, it is not released by neurons in L1CAM-positive exosomes as seen in PD. Alternatively, MSA pathology is mainly confined to the central nervous system whereas Lewy body pathology also affects the enteric or other autonomic neurons early in PD.^2^ Therefore, it is possible that α-synuclein in L1CAM-positive exosomes may arise at least partly from neurons outside the brain. This is not per se a limitation in their utility as a biomarker and further validation of our findings could establish this blood-based marker in the differential diagnosis of PD from MSA, even in the prodromal phase.

Our observations in clinical samples mechanistically suggest that jettison of α-synuclein from neuronal tissues is a specific pathophysiological response across the spectrum of PD that precedes the clinical diagnosis. Accordingly, pSer129 α-synuclein was not consistently detected in neuronal exosomes from blood except in a PD subgroup. Thus, at least in the early stages of disease, exosomal release appears to concern primarily non-pathogenic forms of α-synuclein. PD genetics indicate that protein trafficking to endosomes and lysosomes is relevant to the pathogenic cascade.^36^ Exosomes are derived from intraluminal vesicles within maturing (late) endosomes, also known as multivesicular bodies (MVB). The content of MVB is typically destined for degradation when they fuse with lysosomes or released as exosomes when they fuse with the plasma membrane. It is therefore possible that progressive failure of intraneuronal trafficking from endosomes to lysosomes leads to increased exosomal release of α-synuclein. This model would be consistent with a number of cell-based studies, which showed that α-synuclein is trafficked to endosomes and undergoes lysosomal degradation^37–39^ whereas inhibition of lysosomal function increased α-synuclein release in exosomes in conditioned media.^40 41^ Based on this model, the reported decrease in CSF total α-synuclein in PD^3 4^ could be secondary to an adaptive efflux into serum exosomes in response to defective intraneuronal processing of the protein.

We identified clusterin by mass spectrometry as an abundant protein in neuronal exosomes. Clusterin is a risk gene for Alzheimer’s-type dementia^26 27^ and a stress-induced chaperone in numerous pathological processes. Although total serum clusterin is elevated in AD, this association is controversial^42^ and may involve Aβ-independent pathways.^43^ Recent studies have shown that increased clusterin levels correlate with tau pathology in AD brain^44^ and clusterin expression is protective by reducing TDP-43 or tau aggregation in fly models.^28^ We found that exosomal clusterin levels were elevated in FTD, PSP and CBS, three neurodegenerative diseases that are characterised pathologically by primarily tau or TDP-43 proteinopathy and minimal α-synuclein pathology.^45^ Integration of clusterin quantification with α-synuclein improved the separation of patients with predominantly tau pathology. Because concomitant proteinopathies are frequently found in dementias,^45^ clusterin in combination with α-synuclein could be especially useful in identifying those patients most likely to benefit from therapies targeting intraneuronal α-synuclein.

The finding that neuron-derived exosomal α-synuclein is consistently elevated across populations and remains elevated within individuals with PD when tested in three subsequent visits spanning over a period of 40.3±8.5 months, suggests that measurements of the neuronal exosome content of α-synuclein in serum could be used as a proxy to its intraneuronal processing and thus a potential marker for monitoring disease-modifying therapies that target intraneuronal α-synuclein in brain, especially in the early stages of PD. Given the high risk of RBD conversion to PD^46^ and the wide acceptance of RBD patients as potential candidates for neuroprotective therapies against PD, our study also defined the parameters for an easily accessible, objective readout of underpinning Lewy pathology in this group of prodromal PD. Notably, combined measurements of neuronal exosome content of α-synuclein and clusterin improved the predictive test value of a primary intraneuronal α-synucleinopathy in prodromal PD from an alternative proteinopathy (AUC=0.98) or MSA (AUC=0.94). Therefore, in the right clinical context, assaying of neuron-derived exosomal α-synuclein and clusterin in serum is a predictive blood-based test to differentiate PD from atypical parkinsonism that could be introduced in clinical trials targeting at-risk populations.

## References

[1] Braak H, Del Tredici K, Rüb U, et al Staging of brain pathology related to sporadic Parkinson's disease. Neurobiol Aging 2003;24:197–211. 10.1016/S0197-4580(02)00065-9 12498954

[2] Tofaris GK, Spillantini MG Physiological and pathological properties of alpha-synuclein. Cell Mol Life Sci 2007;64:2194–201. 10.1007/s00018-007-7217-5 17605001 PMC11135986

[3] Mollenhauer B, Locascio JJ, Schulz-Schaeffer W, et al α-Synuclein and tau concentrations in cerebrospinal fluid of patients presenting with parkinsonism: a cohort study. Lancet Neurol 2011;10:230–40. 10.1016/S1474-4422(11)70014-X 21317042

[4] Eusebi P, Giannandrea D, Biscetti L, et al Diagnostic utility of cerebrospinal fluid α-synuclein in Parkinson's disease: a systematic review and meta-analysis. Mov Disord 2017;32:1389–400. 10.1002/mds.27110 28880418

[5] El-Agnaf OMA, Salem SA, Paleologou KE, et al Alpha-Synuclein implicated in Parkinson's disease is present in extracellular biological fluids, including human plasma. Faseb J 2003;17:1–16. 10.1096/fj.03-0098fje 14519670

[6] Shi M, Zabetian CP, Hancock AM, et al Significance and confounders of peripheral DJ-1 and alpha-synuclein in Parkinson's disease. Neurosci Lett 2010;480:78–82. 10.1016/j.neulet.2010.06.009 20540987 PMC2943649

[7] Tofaris GK A critical assessment of exosomes in the pathogenesis and stratification of Parkinson's disease. J Parkinsons Dis 2017;7:569–76. 10.3233/JPD-171176 28922170 PMC5676982

[8] Tomlinson PR, Zheng Y, Fischer R, et al Identification of distinct circulating exosomes in Parkinson's disease. Ann Clin Transl Neurol 2015;2:353–61. 10.1002/acn3.175 25909081 PMC4402081

[9] Shi M, Liu C, Cook TJ, et al Plasma exosomal α-synuclein is likely CNS-derived and increased in Parkinson's disease. Acta Neuropathol 2014;128:639–50. 10.1007/s00401-014-1314-y 24997849 PMC4201967

[10] Cerri S, Ghezzi C, Sampieri M, et al The Exosomal/Total α-synuclein ratio in plasma is associated with glucocerebrosidase activity and correlates with measures of disease severity in PD patients. Front Cell Neurosci 2018;12:125. 10.3389/fncel.2018.00125 29867358 PMC5968118

[11] Szewczyk-Krolikowski K, Tomlinson P, Nithi K, et al The influence of age and gender on motor and non-motor features of early Parkinson's disease: initial findings from the Oxford Parkinson disease center (Opdc) discovery cohort. Parkinsonism Relat Disord 2014;20:99–105. 10.1016/j.parkreldis.2013.09.025 24183678

[12] Hopfner F, Müller SH, Steppat D, et al No association between Parkinson disease and autoantibodies against NMDA-type glutamate receptors. Transl Neurodegener 2019;8:11. 10.1186/s40035-019-0153-0 30984390 PMC6446289

[13] Borroni B, Cosseddu M, Pilotto A, et al Early stage of behavioral variant frontotemporal dementia: clinical and neuroimaging correlates. Neurobiol Aging 2015;36:3108–15. 10.1016/j.neurobiolaging.2015.07.019 26329689

[14] Hughes AJ, Daniel SE, Kilford L, et al Accuracy of clinical diagnosis of idiopathic Parkinson's disease: a clinico-pathological study of 100 cases. J Neurol Neurosurg Psychiatry 1992;55:181–4. 10.1136/jnnp.55.3.181 1564476 PMC1014720

[15] Litvan I, Agid Y, Calne D, et al Clinical research criteria for the diagnosis of progressive supranuclear palsy (Steele-Richardson-Olszewski syndrome). Neurology 1996;47:1–9. 8710059 10.1212/wnl.47.1.1

[16] Armstrong MJ, Litvan I, Lang AE, et al Criteria for the diagnosis of corticobasal degeneration. Neurology 2013;80:496–503. 10.1212/WNL.0b013e31827f0fd1 23359374 PMC3590050

[17] Gilman S, Wenning GK, Low PA, et al Second consensus statement on the diagnosis of multiple system atrophy. Neurology 2008;71:670–6. 10.1212/01.wnl.0000324625.00404.15 18725592 PMC2676993

[18] McKeith IG, Boeve BF, Dickson DW, et al Diagnosis and management of dementia with Lewy bodies. Neurology 2017;89:88–100. 10.1212/WNL.0000000000004058 28592453 PMC5496518

[19] Gorno-Tempini ML, Hillis AE, Weintraub S, et al Classification of primary progressive aphasia and its variants. Neurology 2011;76:1006–14. 10.1212/WNL.0b013e31821103e6 21325651 PMC3059138

[20] Rascovsky K, Hodges JR, Knopman D, et al Sensitivity of revised diagnostic criteria for the behavioural variant of frontotemporal dementia. Brain 2011;134:2456–77. 10.1093/brain/awr179 21810890 PMC3170532

[21] Théry C, Witwer KW, Aikawa E, et al Minimal information for studies of extracellular vesicles 2018 (MISEV2018): a position statement of the International Society for extracellular vesicles and update of the MISEV2014 guidelines. J Extracell Vesicles 2018;7:1535750. 10.1080/20013078.2018.1535750 30637094 PMC6322352

[22] Dalrymple-Alford JC, MacAskill MR, Nakas CT, et al The MoCA: well-suited screen for cognitive impairment in Parkinson disease. Neurology 2010;75:1717–25. 10.1212/WNL.0b013e3181fc29c9 21060094

[23] Postuma RB, Gagnon JF, Vendette M, et al Markers of neurodegeneration in idiopathic rapid eye movement sleep behaviour disorder and Parkinson's disease. Brain 2009;132:3298–307. 10.1093/brain/awp244 19843648

[24] Boot BP, Boeve BF, Roberts RO, et al Probable rapid eye movement sleep behavior disorder increases risk for mild cognitive impairment and Parkinson disease: a population-based study. Ann Neurol 2012;71:49–56. 10.1002/ana.22655 22275251 PMC3270692

[25] Fujiwara H, Hasegawa M, Dohmae N, et al Alpha-Synuclein is phosphorylated in synucleinopathy lesions. Nat Cell Biol 2002;4:160–4. 10.1038/ncb748 11813001

[26] Harold D, Abraham R, Hollingworth P, et al Genome-Wide association study identifies variants at CLU and PICALM associated with Alzheimer's disease. Nat Genet 2009;41:1088–93. 10.1038/ng.440 19734902 PMC2845877

[27] Lambert J-C, Heath S, Even G, et al Genome-Wide association study identifies variants at CLU and CR1 associated with Alzheimer's disease. Nat Genet 2009;41:1094–9. 10.1038/ng.439 19734903

[28] Gregory JM, Whiten DR, Brown RA, et al Clusterin protects neurons against intracellular proteotoxicity. Acta Neuropathol Commun 2017;5:81. 10.1186/s40478-017-0481-1 29115989 PMC5678579

[29] Wang Y, Shi M, Chung KA, et al Phosphorylated α-synuclein in Parkinson's disease. Sci Transl Med 2012;4:121ra20–20. 10.1126/scitranslmed.3002566 PMC3302662 22344688

[30] Jiang C, Alam MT, Silva SM, et al Unique sensing interface that allows the development of an electrochemical immunosensor for the detection of tumor necrosis factor α in whole blood. ACS Sens. 2016;1:1432–8. 10.1021/acssensors.6b00532

[31] Fiandaca MS, Kapogiannis D, Mapstone M, et al Identification of preclinical Alzheimer's disease by a profile of pathogenic proteins in neurally derived blood exosomes: a case-control study. Alzheimers Dement 2015;11:600–7. 10.1016/j.jalz.2014.06.008 25130657 PMC4329112

[32] Kapogiannis D, Boxer A, Schwartz JB, et al Dysfunctionally phosphorylated type 1 insulin receptor substrate in neural-derived blood exosomes of preclinical Alzheimer's disease. Faseb J 2015;29:589–96. 10.1096/fj.14-262048 25342129 PMC4314222

[33] Greffard S, Verny M, Bonnet A-M, et al A stable proportion of Lewy body bearing neurons in the substantia nigra suggests a model in which the Lewy body causes neuronal death. Neurobiol Aging 2010;31:99–103. 10.1016/j.neurobiolaging.2008.03.015 18457903

[34] Colosimo C, Hughes AJ, Kilford L, et al Lewy body cortical involvement may not always predict dementia in Parkinson's disease. J Neurol Neurosurg Psychiatry 2003;74:852–6. 10.1136/jnnp.74.7.852 12810766 PMC1738521

[35] Parkkinen L, Pirttilä T, Alafuzoff I Applicability of current staging/categorization of alpha-synuclein pathology and their clinical relevance. Acta Neuropathol 2008;115:399–407. 10.1007/s00401-008-0346-6 18297293 PMC2270355

[36] Tofaris GK Lysosome-Dependent pathways as a unifying theme in Parkinson's disease. Mov Disord 2012;27:1364–9. 10.1002/mds.25136 22927213

[37] Tofaris GK, Kim HT, Hourez R, et al Ubiquitin ligase Nedd4 promotes alpha-synuclein degradation by the endosomal-lysosomal pathway. Proc Natl Acad Sci U S A 2011;108:17004–9. 10.1073/pnas.1109356108 21953697 PMC3193191

[38] Hasegawa T, Konno M, Baba T, et al The AAA-ATPase Vps4 regulates extracellular secretion and lysosomal targeting of α-synuclein. PLoS One 2011;6:e29460. 10.1371/journal.pone.0029460 22216284 PMC3245276

[39] Alexopoulou Z, Lang J, Perrett RM, et al Deubiquitinase USP8 regulates α-synuclein clearance and modifies its toxicity in Lewy body disease. Proc Natl Acad Sci U S A 2016;113:E4688–97. 10.1073/pnas.1523597113 27444016 PMC4987833

[40] Alvarez-Erviti L, Seow Y, Schapira AH, et al Lysosomal dysfunction increases exosome-mediated alpha-synuclein release and transmission. Neurobiol Dis 2011;42:360–7. 10.1016/j.nbd.2011.01.029 21303699 PMC3107939

[41] Fussi N, Höllerhage M, Chakroun T, et al Exosomal secretion of α-synuclein as protective mechanism after upstream blockage of macroautophagy. Cell Death Dis 2018;9:757. 10.1038/s41419-018-0816-2 29988147 PMC6037700

[42] Yang C, Wang H, Li C, et al Association between clusterin concentration and dementia: a systematic review and meta-analysis. Metab Brain Dis 2019;34:129–40. 10.1007/s11011-018-0325-0 30291488

[43] Slot RER, Kester MI, Van Harten AC, et al ApoE and clusterin CSF levels influence associations between APOE genotype and changes in CSF tau, but not CSF Aβ42, levels in non-demented elderly. Neurobiol Aging 2019;79:101–9. 10.1016/j.neurobiolaging.2019.02.017 31029938

[44] Shepherd CE, Affleck AJ, Bahar AY, et al Intracellular and secreted forms of clusterin are elevated early in Alzheimer's disease and associate with both Aβ and tau pathology. Neurobiol Aging 2019:pii: S0197-4580(19)30389-6. 10.1016/j.neurobiolaging.2019.10.025 31813628

[45] Robinson JL, Lee EB, Xie SX, et al Neurodegenerative disease concomitant proteinopathies are prevalent, age-related and APOE4-associated. Brain 2018;141:2181–93. 10.1093/brain/awy146 29878075 PMC6022546

[46] Postuma RB, Gagnon J-F, Bertrand J-A, et al Parkinson risk in idiopathic REM sleep behavior disorder: preparing for neuroprotective trials. Neurology 2015;84:1104–13. 10.1212/WNL.0000000000001364 25681454 PMC4371408

