# Supplementary material for: Serum neuronal exosomes predict and differentiate Parkinson’s disease from atypical parkinsonism
Source: J Neurol Neurosurg Psychiatry. 2020 Apr 9;91(7):720–9. doi: 10.1136/jnnp-2019-322588 (PMC7361010; doi:10.1136/jnnp-2019-322588)
Supplement: Supplementary data [file jnnp-2019-322588supp001.pdf]

Serum neuronal exosomes predict and differentiate Parkinson's disease from atypical parkinsonism

Cheng Jiang,[1] Franziska Hopfner,[1,2] Antigoni Katsikoudi,[1] Robert Hein,[3] Candan Catli,[3] Samuel Evetts,[1,4] Yongzhi Huang,[5] Hong Wang,[6] John W. Ryder,[6] Gregor Kuhlenbäumer,[2] Gunther Deuschl,[2] Alessandro Padovani,[7] Daniela Berg,[2] Barbara Borroni,[7] Michele T. Hu,[1,4] Jason J. Davis,[3] George K. Tofaris[1*]

Supplementary Material

Synthesis of carboxybetaine methacrylate (CBMA)

CBMA was synthesized according to an adapted literature procedure.[1] 3.16 g DMAEMA (20 mmol, 1 equiv.) was dissolved in 50 mL dry dichloromethane (DCM) and cooled to 0-5 °C with an ice bath. 1.72 g β-propiolactone (24 mmol, 1.2 equiv.) dissolved in 10 mL dry DCM was then added slowly. The solution was stirred at 0-5 °C for 8 h. The resulting white precipitate was isolated by filtration and washed with DCM and Et_2O affording 1.91 g (42%) of pure CBMA. ^1H NMR (400 MHz, D_2O) δ 6.27 – 6.11 (m, 1H), 5.78 (p, J = 1.4 Hz, 1H), 4.65 (dq, J = 7.2, 2.3 Hz, 2H), 3.86 – 3.74 (m, 2H), 3.74 – 3.62 (m, 2H), 3.20 (s, 6H), 2.74 (t, J = 7.9 Hz, 2H), 1.94 (t, J = 1.3 Hz, 3H). Anhydrous DCM was obtained from a MBraun MPSP-800 column and used immediately. NMR spectra (supplementary figure 1) were recorded on a Bruker NMR spectrometer (AVIII HD 400) and referenced to the solvent (δ = 4.79 ppm). 2-(Dimethylamino)ethyl methacrylate (DMAEMA) was obtained from Sigma Aldrich and β-propiolactone was obtained from Alfa Aesar. All chemicals were used as received.

Preparation of poly(carboxybetaine methacrylate) based zwitterionic magnetic beads and antibody conjugation

The magnetic beads were prepared by a two-step approach comprising of the formation of ferrihydrite/formaldehyde composite microbeads and subsequent hydrothermal reduction of the ferrihydrite to Fe_3O_4.[2 3] Poly(carboxybetaine methacrylate), was then formed and coated on the Fe_3O_4 using reversible addition fragmentation chain transfer (RAFT) method to generate pCBMA magnetic beads.[4] Bis(carboxymethyl)trithiocarbonate (Bittc, Sigma) and 4,4′-

Azobis(4-cyanovaleric acid) (ACVA) were used as RAFT agent and initiator, respectively. For conjugation of antibody, the carboxylic acid groups of the pCBMA beads were activated with 2-morpholinoethanesulfonic acid (MES) buffer (50 mM, pH 5.5) containing 50 mg/mL 1-ethyl-3-(3-dimethylaminopropyl) carbodiimide/N-hydroxysuccinimide (EDC/NHS, Sigma) at room temperature for 1 h. Beads were then rinsed with MES buffer and PBS, followed by adding 8 µg of anti-L1CAM (ab80832, Abcam, UK) per 1 mg beads. The mixture incubated on the rotator for 1.5 h at room temperature. The resultant pCBMA-anti-L1CAM beads were washed twice with PBS and used for immunocapture.

Assay development for isolation and detection of neuron-derived exosomes in blood

To specifically isolate exosomes derived from neuronal cells we used an immunoaffinity-based capturing approach with an antibody against the neuronal L1 adhesion molecule (L1CAM) covalently bound to magnetic microbeads. L1CAM belongs to a group of cell adhesion molecules that are primarily expressed in the nervous system and was previously shown to be a surface marker of neuron-derived exosomes isolated from multiple sources, including blood [5]. We further developed this assay to minimise contamination from peripheral sources. To this end, we produced magnetic beads (~2.4 µm) pre-coated with a zwitterionic polymer poly(carboxybetaine methacrylate) pCBMA, via reversible addition fragmentation chain-transfer. Successful polymerisation of pCBMA on beads was shown by attenuated total IR reflection spectroscopy when compared to iron oxide beads (supplementary figure 2A). The antifouling properties of the coated beads were confirmed by reduced non-specific adsorption of bovine serum albumin or total serum protein (supplementary figure 2B and 2C) when compared to commercially available epoxy beads, both conjugated to anti-HA antibodies. The carboxylic acid groups of pCBMA were then activated and cross-linked to anti-L1CAM antibodies and assessed for immunocapture of neuronal exosomes in serum (supplementary figure 3A). Firstly, we showed by SEM that exosomes bound to anti-L1CAM conjugated pCBMA coated beads but not control beads (supplementary figure 3B). Secondly, we tested and confirmed by immunoblotting the presence of both surface (L1CAM, CD81) and internal (syntenin-1, tsg101) exosome markers in lysates of vesicles captured by anti-L1CAM conjugated pCBMA coated magnetic beads (supplementary figure 3C). Thirdly, we profiled the total proteomic composition in L1CAM-captured exosomes from pooled human serum by mass spectrometry and identified 512 proteins. We used gene ontology (GO) term analysis to define enriched functions or components within these proteins. Enrichment scores, the

degree to which a list of proteins in a GO term are represented within the protein list when compared to the total list of proteins tested, were plotted for GO terms that were significant (*p* value threshold of 10^{-3}). The analysis revealed terms enriched in exosomes and related extracellular vesicle functions (supplementary figure 3D). Among the identified proteins were multiple *bona fide* exosome markers such as CD9, syntenin-1, 14-3-3 zeta/delta (YWHAZ), neural cell adhesion protein (N1CAM) as well as the protein clusterin (supplementary figure 3E). For targeted analysis of protein concentration in immunocaptured exosomes we developed a triplex analysis of L1CAM-positive exosomes for total α-synuclein, clusterin and syntenin-1 and demonstrated specific detection of these markers in immunocaptured exosomes (supplementary figure 5).

Fourier tranform infrared- attenuated total reflectance (FTIR-ATR)

Appropriate amount of the prepared pCBMA magnetic beads were wash with ethanol and ultrapure water and dried at 50 °C for FTIR-ATR analysis (Bruker Vertex 80, Bruker Corporation, Ettlingen, Germany). CBMA Monomer and uncoated magnetic beads were used as controls.

Immunoblotting

Immunocaptured exosomes were lysed in LDS buffer (Thermo Fisher) and resolved using sodium dodecyl sulfate polyacrylamide gel electrophoresis (SDS–PAGE), transferred onto polyvinylidene fluoride membranes (PVDF, Invitrogen) and immunoblotted with antibodies against syntenin-1 (ab133267, Abcam), CD81 (sc-5275, Santa Cruz), Tsg101 (ab125011, Abcam) and L1CAM (ab80832, Abcam). All antibodies were used at 1:1,000 dilution. Following incubation with a horseradish peroxidase-conjugated secondary antibody (GE Healthcare) (1:10,000 dilution), chemiluminescence was used for immunodetection (ChemiDoc, Bio-Rad).

SEM

Immunocaptured exosomes were fixed in 2 % glutaraldehyde on clean silicon wafer and washed twice with PBS. After natural evaporation, the samples were coated with around 5 nm platinum

using a sputter coater (Cressington) and imaged with a scanning electron microscope at 5 kV (Zeiss Crossbeam 540).

Mass spectroscopy

Immunocaptured exosomes were lysed in RIPA buffer for 15 min at room temperature. Lysates were reduced using dithiothreitol and alkylated with iodoacetamide. Exosomal proteins were isolated with methanol-chloroform precipitation and digested using 0.1 µg/µL of sequencing grade modified porcine trypsin (Promega) diluted in NH_4HCO_3. Peptides were purified using a C18 spin column (Pierce). The eluted peptides containing acetonitrile were evaporated in a Speedvac (Thermo scientific) to 10 µL and then adjusted to 10 µL with 2% acetonitrile, 0.1% formic acid in ultrapure water. Samples were subsequently analyzed by nanoUPLC-MS/MS using a Waters, nanoAcquity column, 75 µm × 250 mm, 1.7 µm particle size, and a gradient of 1–40% acetonitrile in 60 min at a flow rate of 250 nL/min. Mass spectrometry analysis was performed on a Thermo LTQ Orbitrap Velos (60,000 Resolution, Top 20, CID, Waltham, MA, USA). Raw MS data were analyzed using Progenesis QI for Proteomics software (v3.0; Nonlinear Dynamics, Newcastle upon Tyne, UK). MS/MS spectra were searched against the UniProt Homo Sapiens Reference proteome (retrieved January 6, 2017) using Mascot (v2.5.1; Matrix Science, Inc., Boston, MA), allowing for a precursor mass tolerance of 10ppm and a fragment ion tolerance of 0.05 Da.

Supplementary Figures

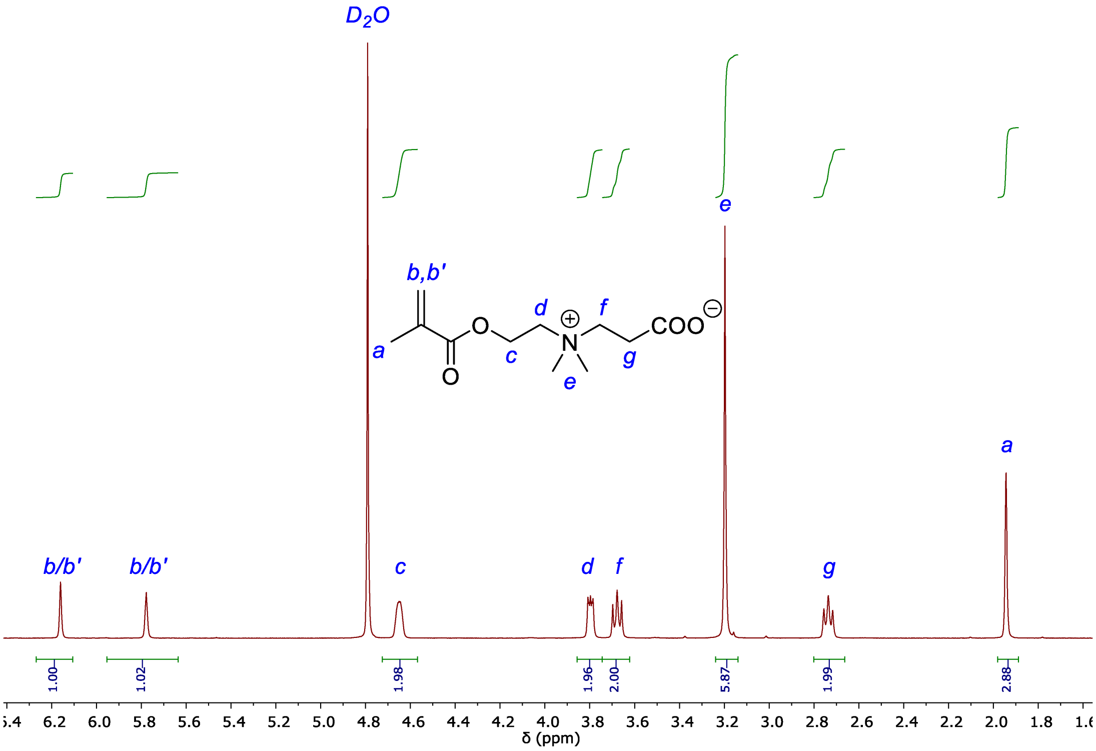

Supplementary Figure 1. Molecular structure of carboxybetaine methacrylate (CBMA) monomer and nuclear magnetic resonance (NMR) spectrum of CBMA in D_2O.

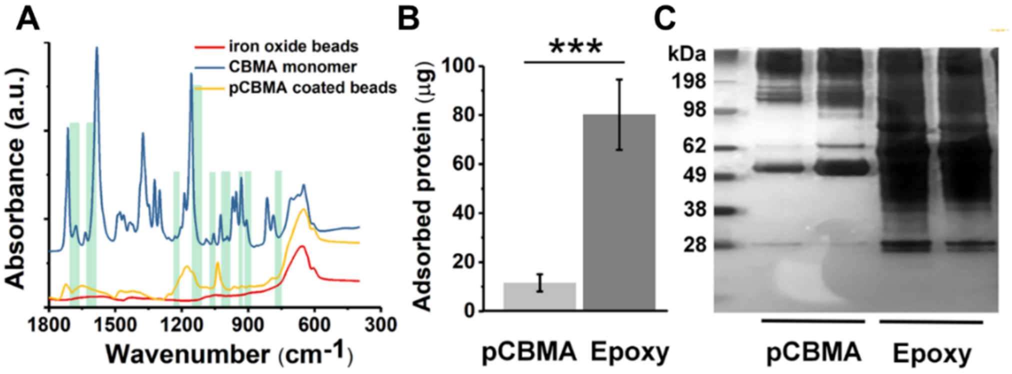

Supplementary Figure 2. (A) FTIR-ATR spectrum of pCBMA coated beads with bare iron oxide beads and CBMA monomer used as controls. Reduced adsorption of BSA **(B)** or serum proteins **(C)** on pCBMA coated beads compared to commercially available epoxy beads, both conjugated to anti-HA antibodies.

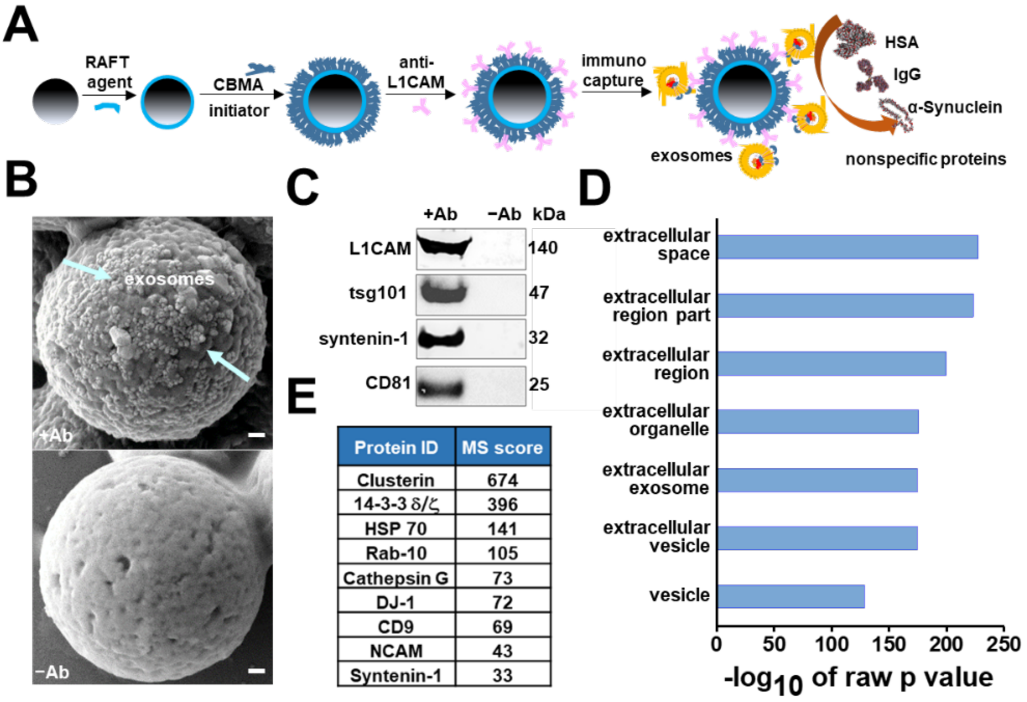

Supplementary Figure 3. pCBMA-based zwitterionic magnetic bead preparation and exosome immunocapture. (A) Synthesis and application of pCBMA coated magnetic microbeads for immunocapture of L1CAM-positive neuronal exosomes in serum. **(B)** SEM of anti-L1CAM conjugated or control pCBMA coated beads demonstrating immunocapture of exosomes from serum (scale bar, 200 nm). **(C)** Lysates of immunocaptured vesicles contain transmembrane (CD81 and L1CAM) and internal exosomal proteins (Tsg101 and Syntenin-1) as shown by immunoblotting. **(D)** GO analysis of proteins identified by mass spectrometry revealed terms enriched in exosomes and related extracellular vesicle functions. **(E)** List of *bona fide* exosomal proteins and top hits identified by mass spectrometry.

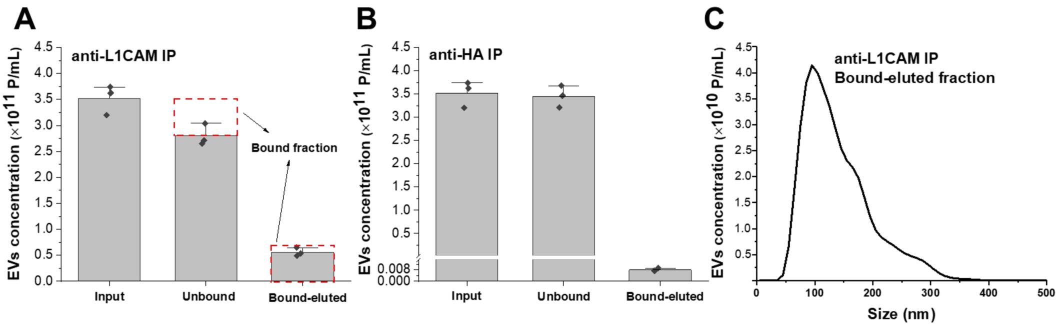

Supplementary Figure 4. NTA analysis of immunocaptured extracellular vesicles. Input, unbound and bound (eluted with pH 2.8 Glycine) fractions during serum exosome immunocapture (n=3). **(A)** Anti-L1CAM coated beads captured a subpopulation of extracellular vesicles as indicated by the drop in the number of particles per ml in the unbound compared to input fraction. **(B)** In anti-HA coated beads, both input and unbound fractions were equal. **(C)** NTA analysis of the eluted extracellular vesicle fraction revealed a peak around 100nm which is consistent with small vesicles of the size of exosomes (typically 40-120nm).

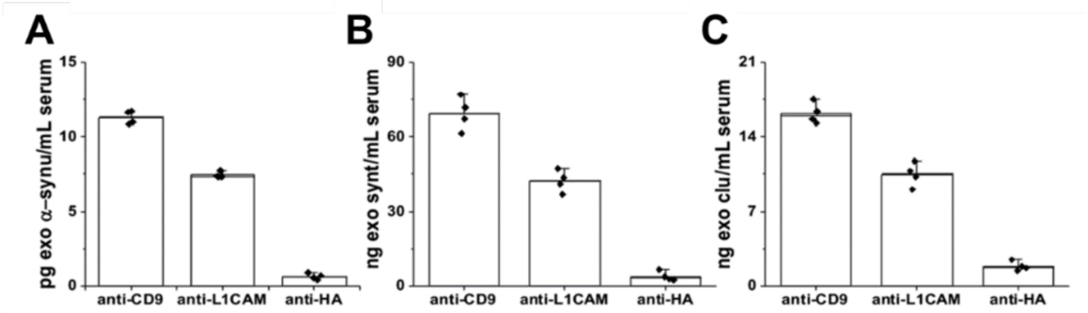

Supplementary Figure 5. Specific detection by triplex electrochemiluminescence of α-synuclein **(A),** syntenin-1 **(B)** and clusterin **(C)** in serum exosomes immunocaptured with anti-CD9 (total exosome population), anti-L1CAM (neuronal exosome subpopulation) or anti-HA (control antibody against epitope not present on exosomes). N=4 biological replicates.

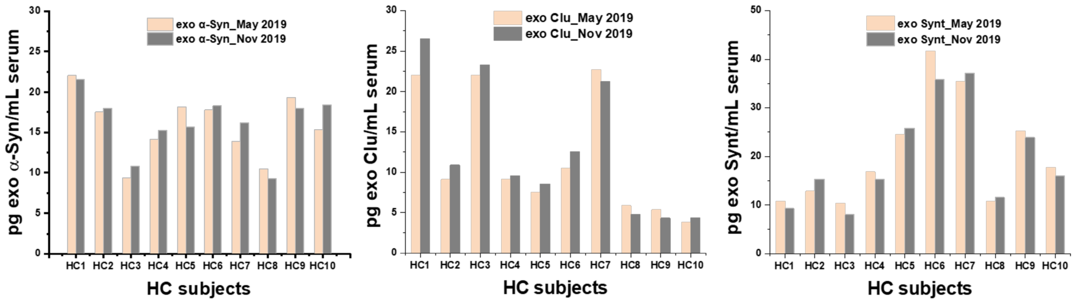

Supplementary Figure 6. Storage does not influence exosomal biomarker levels. A serum sample from each of the 10 individuals shown was frozen after collection and thawed for exosome immunocapture and triplex MSD at two different timepoints 6-month apart. Corresponding protein levels were very similar between tests demonstrating the high reproducibility of the assay.

A

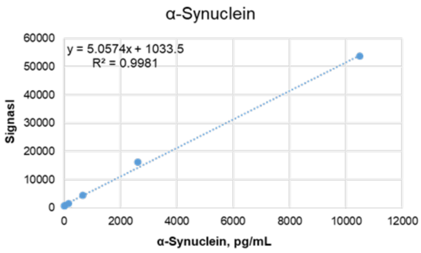

α-Synuclein (pg/mL)	Signal 1	Signal 2	Signal mean	CV	% Recovery mean	Calculated concentrati on mean (pg/mL)	Calculated concentrati on CV
10500	53654	44327	48991	13.5	101	10601	25.7
2625	20314	16222	18268	15.8	103	2698	17.5
656	4013	5003	4508	15.5	95.3	626	16.5
164	2118	1180	1649	40.2	111	181	62.6
41	828	828	828	0.0	73.5	30.2	0.0
10.3	788	728	758	5.6	124	12.8	88.8
2.56	693	715	704	2.21	NA	NA	NA
0	775	714	744	5.79	NA	NA	NA

B

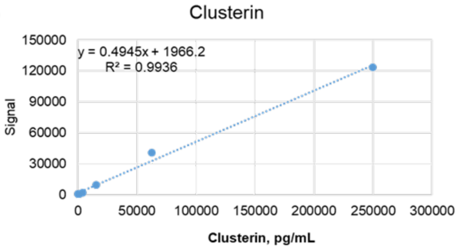

Clusterin (pg/mL)	Signal 1	Signal 2	Signal mean	CV	% Recovery mean	Calculated concentrati on mean (pg/mL)	Calculated concentrati on CV
250000	123552	123592	123592	0.02	93.2	233031	0.04
62500	40318	41746	41032	0.92	104	64874	0.94
15625	9755	9441	9598	2.31	99.5	15553	2.11
3906.3	2422	2142	2282	8.68	98.9	3863	9.54
976.6	902	865	883.5	2.96	100	979	6.57
244.1	607	635	621	3.19	107	262	24.5
0	577	524	550.5	6.81	NA	NA	NA

C

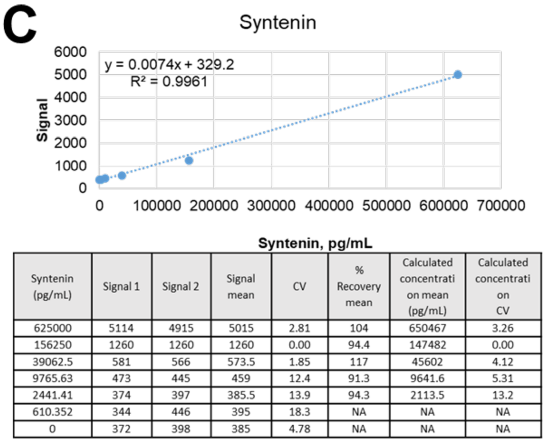

Syntenin (pg/mL)	Signal 1	Signal 2	Signal mean	CV	% Recovery mean	Calculated concentrati on mean (pg/mL)	Calculated concentrati on CV
625000	5114	4915	5015	2.81	104	650467	3.26
156250	1260	1260	1260	0.00	94.4	147482	0.00
39062.5	581	566	573.5	1.85	117	45602	4.12
9765.63	473	445	459	12.4	91.3	9641.6	5.31
2441.41	374	397	385.5	13.9	94.3	2113.5	13.2
610.352	344	446	395	18.3	NA	NA	NA
0	372	398	385	4.78	NA	NA	NA

Supplementary Figure 7. Validation of the triplex assay for detection of exosomal proteins. Validated antibody pairs for α-Synuclein and Clusterin and their standards were purchased from MSD. Antibody pairs for Syntenin-1 and standards were purchased from commercial suppliers (see materials and methods) and validated in-house. The components for three markers were tested and optimised to develop a triplex assay on MSD plates using three different linkers. The calibration curves and dynamic range in the triplexing assay are shown for **(A)** α-Synuclein, **(B)** Clusterin and **(C)** Syntenin-1. Dynamic Range for α-Synuclein: 2.56-10500 pg/mL, lower limit of detection (LLOD): 2.56 pg/mL. Dynamic Range for Clusterin:

244.1-1000000 pg/mL (linear range: 244.1-250000 pg/mL), LLOD: 244.1 pg/mL. Dynamic Range for Syntenin: 610.352-2500000 pg/mL, (linear range: 610.352-625000 pg/mL), LLOD: 610.352 pg/mL.

A

Antibody pairs		standards	
Capture Ab	11A5 (ATCC #PTA822)	α-Syn	pSer129
Detection Ab	4B12 (Biolegend #807802)	human α-Syn (rPeptide)<br>rPeptide S-1001-1	human α-Syn (rPeptide), in vitro phosphorylated by PLK2<br>BE04205-020

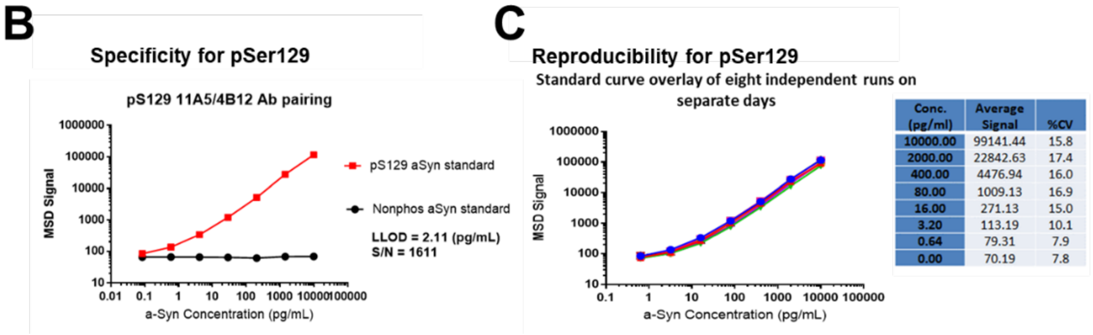

Conc. (pg/ml)	Average Signal	%CV
10000.00	99141.44	15.8
2000.00	22842.63	17.4
400.00	4476.94	16.0
80.00	1009.13	16.9
16.00	271.13	15.0
3.20	113.19	10.1
0.64	79.31	7.9
0.00	70.19	7.8

Supplementary Figure 8. Electrochemiluminescence assay development for the detection of pSer129 α-synuclein. **(A)** Information for antibody pairs used, **(B)** specificity test and **(C)** reproducibility. The lowest limit of detection for pSer129 α-synuclein was 2.11 pg/mL. The lowest detectable concentration based on the calibration curve was 3.2 pg/ml. Proteins in exosomal lysates were 10 times concentrated: 500 µL serum input were used for exosomes capture, and were lysed with 50 µL lysis buffer. Therefore 0.32 pg/mL was considered as a cut-off for detection of exosomal pSer129 α-synuclein in serum to compare results across groups.

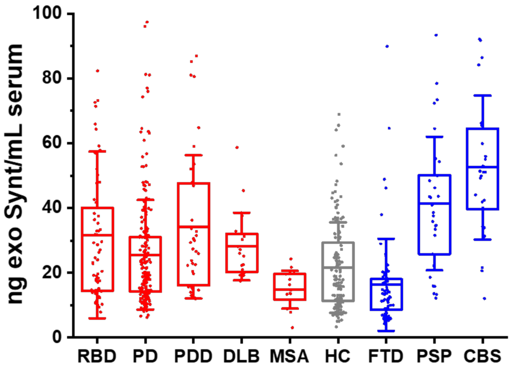

Supplementary Figure 9. Exosomal syntenin-1 levels across disease groups. No disease-specific pattern of distribution was detected across groups that could significantly contribute to biomarker development.

	RBD	PD	PDD	DLB	MSA	HC	FTD	PSP	CBS
Number of individuals	65	230	45	21	14	144	65	35	45
Male (Female)	62 (3)	148 (82)	34 (11)	6 (15)	10(4)	94 (50)	38 (27)	18(17)	27(18)
Male, %	95.4^{a,b,c,d,e,f,g,i}	64.3^{h,i}	75.6^{h,i}	28.6^{a,b,g,h}	71.4^{h,i}	65.2^{h,i}	58.5^{h}	51.4^{h}	60.0^{h}
Age at examination, mean±SD	64.2±8.3^{a,b}	66.3±6.4^{a,b,c,e}	71.5±7.9^{b,c,e,f,h}	68.5±4.9^{b}	68.1±10.8^{b}	60.2±6.2^{a,d,f,h,i}	62.5±7.1^{a,f}	68.0±7.5^{b,e}	61.1±7.2^{a,d,f}
Duration of disease (years)	na	7.7±6.2	8.3±7.8	3.4±3.0	4.9±2.6	na	2.9±2.5	2.8±1.8	1.9±1.3
UPDRS, mean	5.09^{a,d,e,f,g}	24.90^{a,h}	39.58^{d,e,f,h}	20.90	27.7	2.65*	na	24.48	22.49
MoCA, mean	25.52^{a,f,g}	27.32^{a,d,e,g}	18.20^{d,e}	16.27	16.9	26.5*	na	21.40^{e}	22.30

Supplementary Table 1. Demographics of the eight different patient groups from three independent cohorts. Data represent the mean at the time of sample collection. *UPDRS and MoCA were available in 48% of healthy controls. $^{a}p< 0.05$ vs PDD, $^{b}p< 0.05$ vs HC, $^{c}p< 0.05$ vs FTD, $^{d}p< 0.05$ vs PSP, $^{e}p< 0.05$ vs CBS, $^{f}p< 0.05$ vs PD, $^{g}p< 0.05$ vs MSA, $^{h}p<0.05$ vs RBD, $^{i}p<0.05$ vs DLB. Kruskal-Wallis one-way analysis of variance with the Dunn test for post hoc comparison between individual pairings (Age, UPDRS and MoCA scores). Chi-square test was applied to analyse the gender between two groups.

Groups	α-Syn (pg/mL)				Clu (ng/mL)				α-Syn/Clu				Composite of α-Syn and Clu			
	AUC	Cut-off	Spec	Sens	AUC	Cut-off	Spec	Sens	AUC	Cut-off	Spec	Sens	AUC	Cut-off (probability)	Spec	Sens
RBD vs. HC	0.88	14.55	0.72	0.94	–	–	–	–	0.78	2.18	0.74	0.74	0.81	0.20	0.72	0.92
RBD vs MSA	**0.94**	**14.12**	**0.86**	**0.94**	–	–	–	–	0.82	2.15	0.86	0.73	**0.94**	**0.31**	**0.86**	**0.92**
RBD vs. FTD+PSP+CBS	**0.94**	**14.61**	**0.81**	**0.94**	0.83	12.49	0.81	0.72	**0.97**	**1.38**	**0.89**	**0.96**	**0.98**	**0.53**	**0.93**	**0.95**
PD vs. HC	0.86	14.50	0.72	0.81	–	–	–	–	0.77	1.95	0.70	0.73	0.84	0.54	0.73	0.77
PD vs. FTD+PSP+CBS	0.83	14.56	0.81	0.72	0.82	12.06	0.74	0.74	**0.98**	**1.13**	**0.92**	**0.94**	**0.98**	**0.68**	**0.96**	**0.92**
PD+PDD vs. HC	0.85	14.50	0.72	0.81	–	–	–	–	0.77	1.95	0.70	0.72	0.84	0.58	0.70	0.79
PD+PDD vs MSA	0.85	13.70	0.86	0.78	–	–	–	–	0.78	2.14	0.86	0.68	**0.91**	**0.09**	**0.86**	**0.84**
PD+PDD vs. FTD+PSP+CBS	0.85	14.38	0.80	0.74	0.79	12.39	0.71	0.72	**0.97**	**1.13**	**0.92**	**0.94**	**0.98**	**0.41**	**0.96**	**0.94**
RBD, PD, PDD vs. HC	0.82	0.80	0.72	14.50	–	–	–	–	0.76	1.21	0.71	0.73	0.85	0.61	0.71	0.82
RBD, PD, PDD vs. FTD+PSP+CBS	0.86	14.36	0.80	0.83	0.80	12.42	0.73	0.76	**0.96**	**1.39**	**0.89**	**0.89**	**0.96**	**0.79**	**0.97**	**0.92**
RBD, PD, PDD, DLB vs. HC	0.82	14.45	0.75	0.79	–	–	–	–	0.81	2.08	0.76	0.79	0.83	0.65	0.77	0.75
RBD, PD, PDD, DLB vs.FTD+PSP+CBS	0.83	14.32	0.78	0.80	0.87	12.45	0.82	0.85	**0.95**	**1.40**	**0.93**	**0.92**	**0.96**	**0.76**	**0.96**	**0.92**
HC vs FTD+PSP+CBS	–	–	–	–	0.86	12.41	0.81	0.82	0.89	1.41	0.82	0.84	0.89	0.59	0.81	0.86
MSA vs FTD+PSP+CBS	–	–	–	–	**0.93**	**8.99**	**0.93**	**0.90**	**0.94**	**1.22**	**0.94**	**0.93**	**0.97**	**0.83**	**0.94**	**0.93**

Supplementary Table 2. Summary of ROC analyses in patient groups comparing synucleinopathies to controls or other proteinopathies using α-synuclein, clusterin and composite marker (α-synuclein and clusterin). Composite marker was analysed with logistic regression. ROC-based separations were applied where there is significant difference between two groups ($p < 0.001$). High-performance (AUC ≥ 0.90) markers are shown in red.

References

1. Li Y, Liu R, Yang J, et al. Dual sensitive and temporally controlled camptothecin prodrug liposomes codelivery of siRNA for high efficiency tumor therapy. *Biomaterials* 2014;35:9731-45.
2. Deng M, Jiang C, Jia L. N-methylimidazolium modified magnetic particles as adsorbents for solid phase extraction of genomic deoxyribonucleic acid from genetically modified soybeans. *Anal Chim Acta* 2013;771:31-36.
3. Zhang L, Chen L, Wan Q-H. Preparation of Uniform Magnetic Microspheres through Hydrothermal Reduction of Iron Hydroxide Nanoparticles Embedded in a Polymeric Matrix. *Chem Mater* 2008;20:3345-53.
4. Ratcliffe LP, Blanazs A, Williams CN, et al. RAFT polymerization of hydroxy-functional methacrylic monomers under heterogeneous conditions: effect of varying the core-forming block. *Polym Chem* 2014;5:3643-55.
5. Shi M, Liu C, Cook TJ, et al. Plasma exosomal alpha-synuclein is likely CNS-derived and increased in Parkinson's disease. *Acta Neuropathol* 2014;128:639-50.
